# Accuracy of surface‐guided patient setup for conventional radiotherapy of brain and nasopharynx cancer

**DOI:** 10.1002/acm2.13241

**Published:** 2021-03-31

**Authors:** Sang Kyu Lee, Sheng Huang, Lei Zhang, Ase M. Ballangrud, Michalis Aristophanous, Laura I. Cervino Arriba, Guang Li

**Affiliations:** ^1^ Department of Medical Physics Memorial Sloan Kettering Cancer Center New York NY USA

**Keywords:** brain and head‐and‐neck cancer, cone‐beam CT, optical surface imaging, radiotherapy treatment planning, tattoo‐less patient setup

## Abstract

**Purpose:**

To evaluate the accuracy of surface‐guided radiotherapy (SGRT) in cranial patient setup by direct comparison between optical surface imaging (OSI) and cone‐beam computed tomography (CBCT), before applying SGRT‐only setup for conventional radiotherapy of brain and nasopharynx cancer.

**Methods and Materials:**

Using CBCT as reference, SGRT setup accuracy was examined based on 269 patients (415 treatments) treated with frameless cranial stereotactic radiosurgery (SRS) during 2018‐2019. Patients were immobilized in customized head molds and open‐face masks and monitored using OSI during treatment. The facial skin area in planning CT was used as OSI region of interest (ROI) for automatic surface alignment and the skull was used as the landmark for automatic CBCT/CT registration. A 6 degrees of freedom (6DOF) couch was used. Immediately after CBCT setup, an OSI verification image was captured, recording the SGRT setup differences. These differences were analyzed in 6DOFs and as a function of isocenter positions away from the anterior surface to assess OSI‐ROI bias. The SGRT in‐room setup time was estimated and compared with CBCT and orthogonal 2D kilovoltage (2DkV) setups.

**Results:**

The SGRT setup difference (magnitude) is found to be 1.0 ± 2.5 mm and 0.1˚±1.4˚ on average among 415 treatments and within 5 mm/3˚ with greater than 95% confidence level (*P* < 0.001). Outliers were observed for very‐posterior isocenters: 15 differences (3.6%) are >5.0mm and 9 (2.2%) are >3.0˚. The setup differences show minor correlations (|r| < 0.45) between translational and rotational DOFs and a minor increasing trend (<1.0 mm) in the anterior‐to‐posterior direction. The SGRT setup time is 0.8 ± 0.3 min, much shorter than CBCT (5 ± 2 min) and 2DkV (2 ± 1 min) setups.

**Conclusion:**

This study demonstrates that SGRT has sufficient accuracy for fast in‐room patient setup and allows real‐time motion monitoring for beam holding during treatment, potentially useful to guide radiotherapy of brain and nasopharynx cancer with standard fractionation.

## 1 INTRODUCTION

Surface‐guided radiotherapy (SGRT) using optical surface imaging (OSI), as a special form of image‐guided radiotherapy (IGRT), has been increasingly applied to guide patient setup and monitor patient motion during treatments, such as cranial frameless stereotactic radiosurgery (SRS) and left‐sided breast deep‐inspiration breath‐hold (DIBH) treatment.^1^, ^2^, ^3^ The advantages of SGRT include nonionization radiation 3D imaging with the patient’s external anatomy and real‐time 4D imaging for motion tracking and threshold gating. Most clinical applications involve patient setup and motion monitoring either for rigid anatomy with a fixed relationship between the skin surface and deep‐seated lesions, such as brain cancer,^4^, ^5^, ^6^, ^7^ or for superficial lesions, such as breast cancer.^8^, ^9^, ^10^, ^11^ For patient setup, the external body contour from the planning CT is mostly used as the reference, while for motion monitoring, an on‐site surface reference is usually captured, excluding the residual setup error. The OSI has recently been applied to achieve tattoo‐free SGRT patient setup and motion monitoring as a replacement of conventional tattoo‐laser alignment setup.^10^, ^11^ Other clinical applications may include SGRT for deformable anatomy with external‐internal motion modelling,^12^, ^13^, ^14^ patient‐gantry collision detection during radiotherapy,^15^, ^16^ and patient identification and registration via facial recognition.^17^

For brain and head‐and‐neck (HN) patients treated with conventional fractionation, room lasers are often used to align the patient with native anatomic landmarks, such as the nose, eyes, and tragus, as well as the tattoos, bb’s, or cast lines on the thermoplastic masks.^18^ Due to the large setup uncertainties, the safety margin for partial brain and HN setups are usually 5–6 mm based on 2 CBCT studies^19^, ^20^ and 3–10 mm for conventional setup and 3–6 mm using helical tomotherapy CT for setup in another study.^21^ Wang et al. studied 22 patients with 505 CBCT setups and concluded 5–6 mm safety margin for conventional setup and 3 mm safety margin for CBCT setup.^19^ Leitzen et al. studied 15 HN patients using mega‐voltage CT (MVCT) for IGRT setup and evaluated the setup margin of 3–5 mm for MVCT‐based setup and 3–10 mm for conventional setup.^21^ Gopan and Wu evaluated SGRT setup accuracy in 11 HN patients with simulated OSI patient surface contours from 77 helical computed tomography (CT) images including planning CT during their 6‐week treatment courses.^22^ They found that the setup uncertainty less than 5 mm had 90% confidence level and higher setup uncertainties occur as the site is further away from the skull due to the deformable cervical spine. However, as the study does not involve OSI imaging, it was not under clinical conditions as any interference items, such as facial masks, were excluded. Kuo et al. reported a phantom study using CBCT, on‐board and ceiling‐floor‐mounted 2DkV, as well as OSI based on both anatomic‐based and point‐based registration.^23^ Both isocenter and target registration errors were reported and a maximum of 2.5 mm OSI uncertainty was found. To apply SGRT‐only patient setup in the clinic to treat brain and HN patient with standard fractionation, a thorough evaluation of SGRT setup accuracy and other advantages is needed with a large clinical dataset.

In this study, we investigated 415 treatments of 269 brain patients who were set up with both OSI and cone‐beam CT (CBCT) for the SRS treatments, and the SGRT setup differences were acquired prospectively in 2018–2019 and evaluated retrospectively in 6 degrees of freedom (DOF) using CBCT as the reference. In our clinical protocol, we specifically requested to capture an OSI verification image immediately after CBCT setup, so that it was possible to assess SGRT setup differences through direct comparison. Due to registration landmark differences in these two imaging modalities, the SGRT setup differences were evaluated as a function of the distance of the isocenter to the skin surface, in addition to generic statistical analyses. The objective of this study is to assess the feasibility, accuracy, and time requirement of SGRT‐only setup without skin markers, daily 2DkV, or CBCT for radiotherapy of brain or nasopharynx cancer with the standard fractionation.

## 2 METHODS AND MATERIALS

In this study, 415 treatment setups of 269 patients with both OSI and CBCT were analyzed. A treatment protocol was designed with initial SGRT, then CBCT setup, followed immediately by capturing a verification AlignRT image, illustrating the difference between OSI and CBCT. Some earlier‐treated patients also received 2DkV verification until this step was removed from the protocol. These patients were treated with brain SRS from 2018 to 2019 using a CDR head immobilization device on the CDR couch extension (CDR, Calgary, Canada) attached to a PerfectPitch couch in 6 degrees of freedom (DOF) of a TrueBeam machine with HD multileaf collimators (Varian, Palo Alto, CA). Before the patient entered the room, the treatment isocenter was determined based on 3 ball‐bearing (BB’s) on the open‐face mask and the setup instruction with the isocenter center shifts from the reference point using the room lasers. Then, the couch position was acquired to facilitate the setup. The OSI system was AlignRT (VisionRT, London, UK), which was used for initial SGRT real‐time patient setup against the external body contour of planning CT, capturing a verification image after CBCT setup for comparison, and acquiring a new on‐site reference image for motion monitoring during SRS treatments. Therefore, SGRT setup uncertainty and speed can be evaluated via direct comparison with the CBCT setup and timing, respectively. The region of interest (ROI) was drawn within the open‐face mask area above the lips on the external contour of the planning CT. The goal is to use the SRS patient dataset to estimate SGRT‐only setup accuracy for radiotherapy of brain and nasopharynx cancer with conventional fractionation.

### 2.1 Description of patient data acquired from SGRT and IGRT stereotactic radiosurgery

In the SRS treatment procedure, all patients were initially set up inside the room with 6DOF SGRT guidance in real‐time delta (RTD) mode. After a patient was positioned into the CDR immobilization device, the patient’s head rotation was first corrected by adjusting head position and then the Pitch/Roll knobs of the CDR couch extension after the open‐face mask placement, followed by translational correction with couch shift. Before closing the door for CBCT, the RTD was turned on to monitor patient motion during CBCT, CBCT/CT image registration, and setup verification from a physicist and approval by a physician. This process also warmed up the AlignRT system in the RTD mode to eliminate the baseline‐drift error,^3^, ^5^, ^7^ which produces an error of ~0.3 mm due to the thermal heat in the camera systems but stabilized after 5‐ to 10‐minute RTD.

After CBCT/CT registration in 6DOF, the shifts were applied from the console using the 6DOF couch. Because of the use of initial SGRT setup, CBCT shifts were small, usually within 2 mm and 1˚ in any DOF. Immediately after CBCT shifts were applied, an OSI verification image was captured, recording the difference between OSI and CBCT. These differences for all 415 treatments (269 patients) were used to evaluate SGRT‐only setup accuracy. Then, an on‐site OSI reference image was captured for motion monitoring during treatment. The time duration of SGRT setup was saved in the patient’s data folder and the CBCT/2DkV SRS patient setups and treatments were recorded in the ARIA Offline Review (Varian, Palo Alto, CA). The data were used to estimate the time spending and saving for the SGRT‐only setup.

### 2.2 SGRT setup uncertainty assessment and statistical analyses

Various statistical analyses were performed, including SGRT setup‐difference distribution and correlation among the 6DOFs. Significance level (α) of 0.05 was used for rejecting the null hypothesis and was adjusted for multiple comparisons when applicable using the Bonferroni method.^24^ Statistical significance of difference among group means was tested using a Student’s t‐test for 2 groups and Analysis of Variance for more than 2 groups. These tests were performed using R version 3.6.1. Correlation between translational and rotational shifts was assessed based on the correlation calculation among the 6DOF.

The dependency of SGRT setup differences on the isocenter location was assessed because the OSI ROI had a bias on the anterior surface, unlike the skull landmark that “evenly” spreads in all directions of the head in CBCT‐to‐planning‐CT registration. The location of the registration landmark was a major difference between the OSI and CBCT. Therefore, we hypothesize that there is a dependency of SGRT setup differences with the isocenter vertical location: the farther away from the anterior ROI, the larger the setup difference would become.

For simplicity, the 6DOF residual OSI differences were summarized into 2 translational and rotational components by taking the magnitude (MAG) of the translational and rotational vectors with 3 components:

(1) $${\text{MAG}}_{\text{trans}}=\Delta_{\text{trans}}=\sqrt{\Delta_{\text{AP}}^{2}+\Delta_{\text{SI}}^{2}+\Delta_{\text{LR}}^{2}}$$

(2) $${\text{MAG}}_{\text{rot}}=\Delta_{\text{rot}}=\sqrt{\Delta_{\text{yaw}}^{2}+\Delta_{\text{roll}}^{2}+\Delta_{\text{pitch}}^{2}}$$

where AP is the anterior‐posterior (or vertical, VRT) direction and axis for the yaw rotation, SI is the superior‐inferior (or longitudinal, LNG) direction and axis for the roll rotation, and LR is the left‐right (or lateral, LAT) direction and axis for the pitch rotation. Note that the MAG_rot_ is purely a vector magnitude, similar to MAG_trans_, but does not have associated physical meaning. The location of the isocenter was categorized in two ways: First, using the brainstem as a reference, 3 zones were defined in the AP and LR directions and 2 zones in the SI direction. Second, the plans were grouped into four ranges of skin‐to‐isocenter distance, which was defined by projecting the isocenter to the midplane in the LR direction (laterally “centralized” isocenter) and measuring the distance to the anterior surface along the lateral midplane. Using these 2 methods, the anterior ROI was assessed for potential setup bias in any directions.

### 2.3 SGRT‐only patient setup time and SGRT real‐time motion monitoring

The time for SGRT‐only patient setup should be the same as pre‐CBCT SGRT setup in SRS treatment. The SGRT setup time was recorded in the first RTD guidance, which often started with a large 6DOF setup error that decreased gradually to sub‐mm and sub‐degree, indicating the completion of the SGRT‐only patient setup. A long RTD motion monitoring during the CBCT and registration should follow. The IGRT setup time was dissected as scanning time, registration time, and waiting time for MD attending approval. The times for both SGRT setup using OSI and IGRT setup using CBCT and 2DkV were analyzed.

Of the 415 treatments, there were 14 incidents of movement (3.4%) from 13 patients detected by AlignRT RTD motion monitoring during beam delivery. If the motion was detected at a couch rotation angle, our procedure was to move the couch back to zero position and capture another SGRT verification image to verify if it was caused by patient motion (∆>1.0 mm or 1.0˚) or it was a false positive (∆<1.0 mm or 1.0˚) due to the AlignRT couch‐angle dependency error.^25^, ^26^ If indeed a patient moved away from the setup position by >1.0 mm, the patient would be set up again using CBCT. These incidents were recorded in the ARIA offline review as a CBCT scan between the first and the last beam delivery.

## 3 RESULTS

### 3.1 SGRT uncertainties in 6DOF and mild translation‐rotation correlations

The 6DOF differences between OSI and CBCT are referred to as the SGRT setup difference in the 415 setups of 269 patients, as tabulated in Table 1. The average and magnitude of the differences are 0.5 ± 1.4 mm and 1.0 ± 2.5 mm in translation and 0.1˚±0.8˚ and 0.1˚±1.4˚ in rotation, respectively. The confidence level for *MAG_trans_* < 5 mm is 97% and for *MAG_rot_* < 3˚ is 98%.

**Table 1:** Translational and rotational differences between SGRT and cone‐beam computed tomography (CBCT) in and around the anteroposterior (AP, or VRT), superior‐inferior (SI, or LNG), and left‐right (LR, or LAT) directions, respectively. The data show the SGRT setup uncertainties using facial ROI beyond CBCT using the skull as the registration landmark.

Parameters	Translational differences (mm)					Rotational differences (degree)				
	AP	SI	LR	Mean	MAG^a^	Yaw	Roll	Pitch	Mean	MAG^a^
Average	0.2	1.0	0.3	0.5	1.0	0.1	0.0	0.0	0.1	0.1
St. Dev	1.2	2.0	0.9	1.4	2.5	0.7	0.5	1.1	0.8	1.4
Maximum	7.4	9.4	6.0	7.6		2.2	1.6	3.4	2.4	
Minimum	−4.4	−7.5	−2.4	−4.8		−3.2	−2.4	−3.8	−3.1	

^a^ MAG is an abbreviation of magnitude [Eqs. (1) and (2)] of the translational and rotational vectors.

**Fig. 1:**
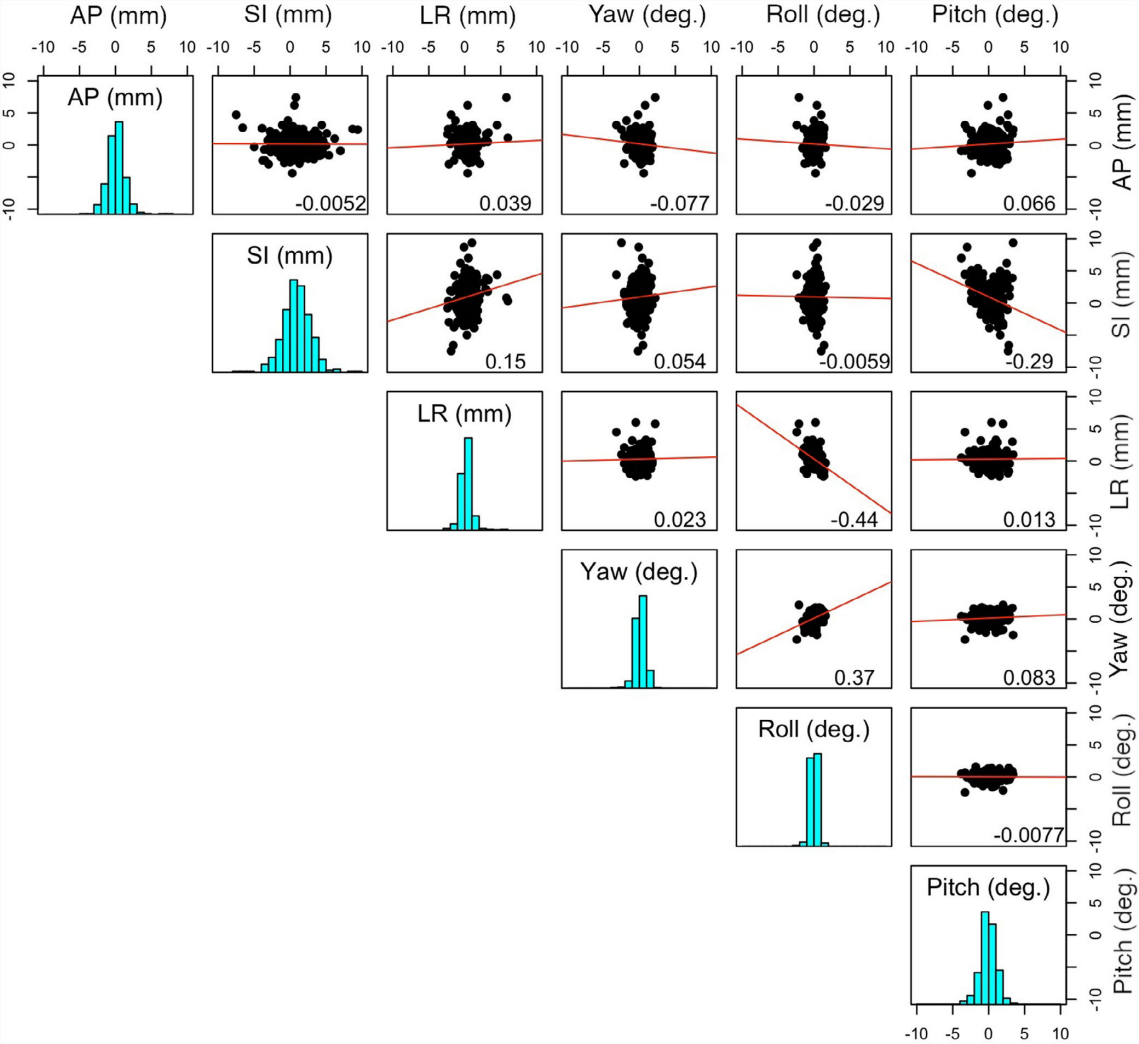
Distributions of the SGRT setup differences in reference to CBCT and their pairwise relationships among 6 degrees of freedom (DOFs). The 3 translations (mm) are anterior‐posterior (AP), superior‐inferior (SI), and left‐right (LR), and the 3 rotations (˚) are Yaw (around an AP axis), Roll (around an SI axis), and Pitch (around an LR axis). The diagonal cells display the setup difference distributions (histogram) in the 6DOFs. The 2D scatter distribution plots with the 2 corresponding DOFs, together the best linear fits and the Pearson’s correlation coefficients, are shown in upper triangle panels.

The difference distribution plots, together with correlation coefficients and linear fits, are shown in the box plot (Fig. 1). In this format, the difference distributions with linear fits are displayed with the correlation coefficients in the upper‐right triangle. Interestingly, some mild correlations were observed: r = −0.44 between LR and Roll (around the SI axis through the isocenter) and r = −0.29 between SI and Pitch (around the LR axis through the isocenter), suggesting possible ambiguity in the 6DOF surface registration results: either translational or rotational shifts may achieve the similar alignment. The translational difference along the SI direction showed the widest spread around the mean among the 3 translations. The pitch difference, which is mildly correlated with the SI difference, also showed the largest deviation from the mean among the rotational components. A mild correlation (0.37) between yaw and roll rotations was also observed.

### 3.2 SGRT setup differences in relationship with the vertical distance of “centralized” isocenter

**Fig. 2:**
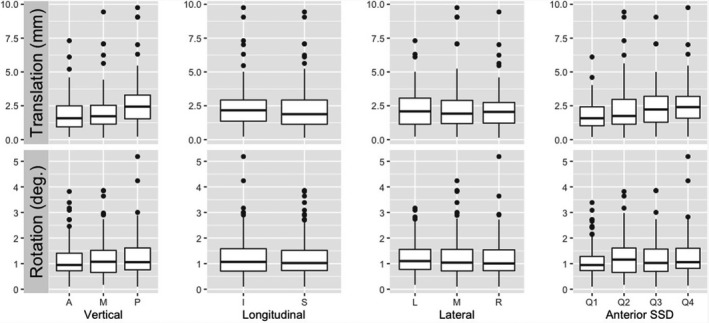
Boxplots for distributions of translational and rotational differences in isocenter zones and anterior SID (skin‐to‐isocenter distance). The median and 25%–75% percentile are shown in a box, together with outliers (dots), which are associated with very large SID, away from the anterior region of interest (ROI).

**Fig. 3:**
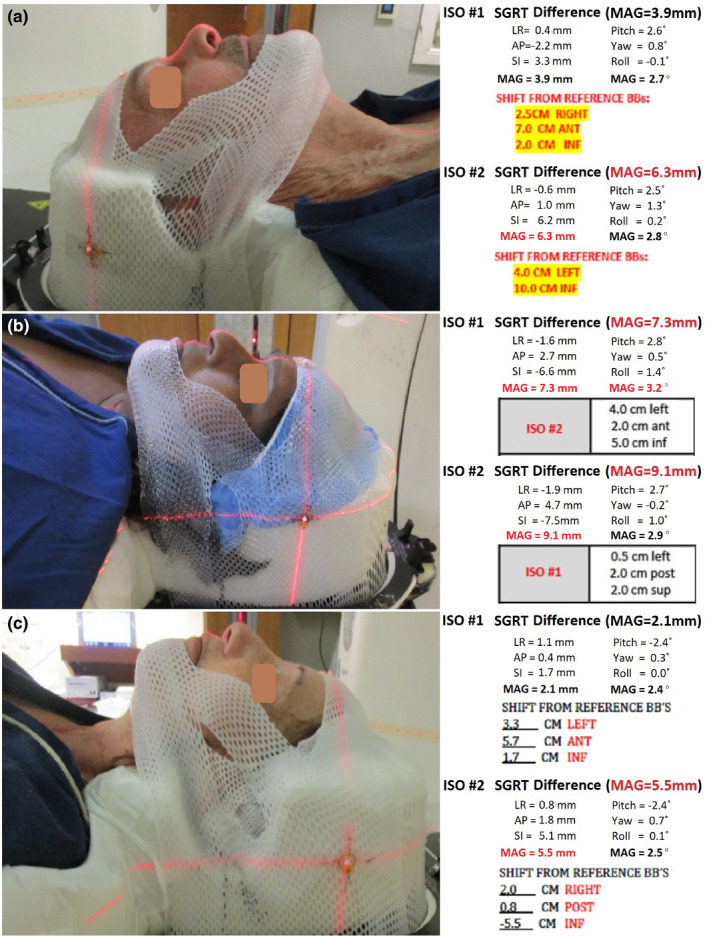
Examples of 4 outliers (>5 mm) and the isocenter dependency of SGRT setup differences. The AP shifts from the BB’s determine isocenter vertical positions (no AP shift means at the BB’s). In patients (a) and (c), the differences are normal for ISO #1 (at the mid brain), but the differences are outliers for ISO #2 (at the posterior brain). In (b), both ISOs are outliers as the isocenters are very posterior. Note 1: The largest differences are in SI translation and Pitch rotation in these cases. Note 2: the ISO #2 has zero AP shift in case (a).

Table 2 and Fig. 2 illustrate the setup differences as the function of the isocenter location in 3 directions. The setup difference does not vary significantly with the location of the isocenter, except for the translational difference with AP location. Table 3 shows a clear increasing trend of the mean translational setup difference as the vertical distance increases between the skin and the laterally centralized isocenter. The setup difference increases with the vertical skin‐to‐isocenter distance (*P* < 0.001) is significant and indicates the bias of the ROI location in the SGRT setup, supporting the hypothesis that SGRT setup difference is skin‐to‐isocenter distance dependent, although the dependency is mild. Interestingly, almost all 15 outliers are associated with very large vertical skin‐to‐isocenter distance, and 4 outliers are illustrated in Fig. 3. In addition, in Figs. 3(a) and 3(c), the patients were treated with 2 isocenter for multiple lesions, and only at the posterior isocenters (ISO #2) SGRT setup yields outliers. Fig. 3(b) shows 2 outliers as both ISOs are posteriorly located in the cerebellum.

**Table 2:** Relationships between the mean SGRT translational/rotational difference from CBCT and isocenter location. The magnitude of the translational ($\Delta_{\text{trans}}$) and rotational ($\Delta_{\text{rot}}$) differences were used [defined in Eqs. (1) and (2)].

Isocenter location		Translation difference (mm)		Rotation difference (˚)	
		Average	St. dev	Average	St. dev
AP^b^	Anterior	1.8	1.3	1.2	0.8
	Medial	2.0	1.4	1.2	0.8
	Posterior	2.6	1.5	1.2	0.7
	*P*‐value^a^	**<0.001**		0.96	
SI^b^	Superior	2.4	1.5	1.2	0.7
	Inferior	2.2	1.5	1.2	0.7
	*P*‐value^a^	0.26		0.88	
LR^b^	Left	2.4	1.4	1.15	0.7
	Medial	2.3	1.5	1.07	0.8
	Right	2.2	1.4	1.06	0.7
	*P*‐value^a^	0.71		0.56	

SGRT, surface‐guided radiotherapy; CBCT, cone‐beam computed tomography.

Bold emphasizes the *P*‐value.

^a^ The *P*‐value is calculated using a two‐sample t‐test (SI) or analysis of variance (AP. LR).

^b^ The brainstem is used as the reference to separate the brain into different sections.

**Table 3:** Relationships between the mean SGRT translational/rotational differences and the vertical depth from isocenter to the anterior surface at the midline of the brain. The magnitude of the translational ($\Delta_{\text{trans}}$) and rotational ($\Delta_{\text{rot}}$) differences were used [defined in Eqs. (1) and (2)].

Vertical depth of isocenter at the midline of the brain (cm)^a^	Translational difference (mm)		Rotational difference (˚)	
	Average	St. dev	Average	St. dev
Q1: <8.85	1.8	1.0	1.1	0.7
Q2: 8.85–11.8	2.3	1.7	1.3	0.8
Q3: 11.8–14.3	2.4	1.5	1.2	0.7
Q4: >14.3	2.6	1.5	1.2	0.8
*P*‐value^b^	**<0.001**		0.32	

Bold emphasizes the *P*‐value.

^a^ The vertical skin‐to‐isocenter distance is obtained by first shifting the isocenter laterally to the midline. This further analysis confirms the initial results in Table 2.

^b^ The *P*‐value is calculated for differences in means across four depth ranges (Q1 to Q4) using the Analysis of Variance.

### 3.3 Patient movement during treatment observed by SGRT motion monitoring

**Fig. 4:**
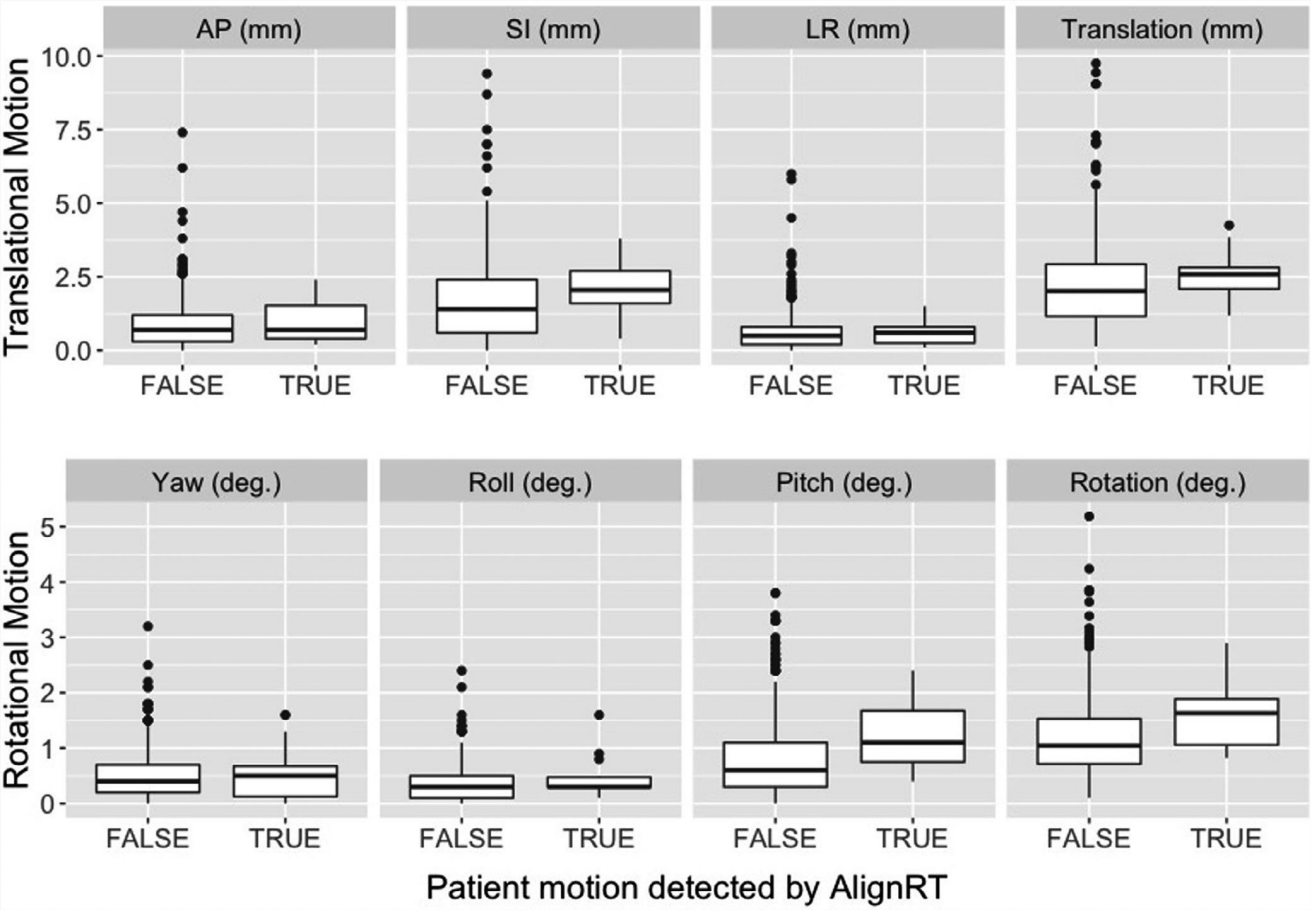
SGRT setup differences between the treatments without motion beyond the threshold (FALSE) and the treatments with patient motion out of tolerance (TRUE). Note that the action thresholds at a couch angle are 1.5 mm and 1˚, in which the enlarged translational threshold is to account for the couch angle dependency error from an AlignRT system. The median and 25%–75% percentile are shown in a box, together with outliers (dots) due to very‐posterior isocenters.

Figure 4 shows the SGRT motion monitoring catches 14 treatments (3.4%), in which patients moved out of the pre‐established tolerance (1.0 mm at couch zero and 1.5 mm at other couch rotations) during treatment and a re‐setup was conducted to correct the motion. There was a noticeable difference in the pitch and SI setup differences, but not statistically significant due to a small number of incidences. It is worthwhile to mention that there is a large difference in the pitch setup, suggesting that the head “nodding” motion may be the primary cause of patient motion during treatment. These motion outliers occur infrequently with relatively small motion (<2 mm beyond the SRS tolerance).

### 3.4 SGRT‐only setup time comparing with CBCT and 2DkV setup times

Table 4 tabulates the patient setup times using various imaging modalities, including SGRT, CBCT, and 2DkV. The SGRT‐only setup time is 0.8 ± 0.3 mins, while CBCT and 2DkV setup times are much longer, including the waiting time for a physician to approve the setup. The time variations are quite large owing to the patient‐specific and/or physician‐specific matters.

**Table 4:** Clinical setup time (in minutes) using SGRT, CBCT, and 2DkV for brain patients.

Clinical action	Time (in minutes)		
	Average	St. Dev	Range
Total setup	11.8	5.2	4.5–47.7
SGRT (including patient positioning)	0.8	0.3	0.5–2.0
CBCT (imaging + registration)	3.4	3.8	0.9–7.3
2DkV Pair (imaging + registration)	1.1	0.6	0.7–4.4
Waiting (physician approval)	6.7	‐	1.1–25.9
Total treatment	27.6	5.7	14.2–82.2
No. of lesions	2	‐	1–8
No. of beam arcs	3.9	‐	3–10

SGRT, surface‐guided radiotherapy; CBCT, cone‐beam computed tomography; 2DKV, 2D kilovoltage.

## 4 DISCUSSION

### 4.1 SGRT‐only setup for non‐SRS brain and nasopharynx treatments

The objective of this study is to assess the accuracy of the SGRT‐only patient setup that can be applied to improve patient setup over the conventional setup in radiotherapy of brain and nasopharynx cancer with standard fractionation. The assessment of uncertainty of the SGRT‐only setup is through direct determination by the SGRT 6DOF shifts right after a patient has been set up based on the CBCT‐to‐planning‐CT registration. Therefore, this method minimizes the possibility of patient motion between the 2 imaging scans. It is worthwhile to mention that as CBCT (used as the reference) may have sub‐mm uncertainty,^2^ the overall SGRT uncertainty should be the sum of the CBCT setup uncertainty and SGRT differences.

In addition to many other differences between OSI and CBCT, the location of their ROI is a major assessable difference. Because SGRT surface registration is based on the ROI, which is on the anterior facial surface of the patient’s head, there is a bias toward the anterior anatomy, unlike the skull landmark in CBCT‐to‐planning‐CT registration. SGRT only aligns to the partial anterior surface of the head and the posterior alignment is unknown. This triggered us to make the hypothesis that the SGRT setup difference is dependent of the isocenter, meaning the farther away from the anterior surface ROI, the larger the setup difference. In fact, this study has illustrated that the SGRT setup difference is a function of the vertical skin‐to‐isocenter distance. The results in Table 3 and Fig. 2 suggest that the SGRT setup difference is linearly increasing as the vertical distance increases. However, the differences between most anterior and most posterior zones are mild, less than 1.0 mm, implying that the anterior bias of the ROI is unlikely to be as clinically impactful as initially thought. Other than the AP direction, we did not find any significant trend of dependency on the isocenter location.

It is worthwhile to mention that a mild negative correlation was observed between SI translation and pitch rotation (r = −0.29) and between LR translation and roll rotation (correlation = −0.44), suggesting possible ambiguity in surface registration results. This means that a translation shift may be replaced by the corresponding rotational shift, resulting in almost equally well‐registered surfaces. Interestingly, a mild correlation (r = 0.37) between Yaw and Roll rotations is also shown in Fig. 1. In the clinic, such phenomena may have been observed and this study provides the quantitative analysis of the observation.

### 4.2 Clinical benefits for SGRT‐only setup and option for motion monitoring

In IGRT patient setup using CBCT and 2DkV, it requires staff to leave the room and close the door, making it impossible to adjust the patient position. Although CBCT and 2DkV align with the internal bony structures, they do give the patient extra radiation dose, take longer time to scan, and require physician for approval before treatment. In contrast, SGRT patient setup is performed inside the room, the surface image is registered automatically, and the setup does not require the physician to approve, therefore, the treatment may start as soon as the automatic alignment meets the clinical criteria. However, as demonstrated in this study, the SGRT setup uncertainty has a magnitude of 1.0 ± 2.5 mm and 0.1˚±1.4˚ and is within 5mm/3˚ at >95% confidence level, and SGRT‐only setup is usually less than 1 min to complete, reduced by one‐order of magnitude. Compared with previously reported conventional setup accuracy, SGRT provides an advantage while the setup time is roughly the same.

Within the OSI ROI, the skin deformation is limited, unless a patient experiences substantial weight changes, including weight loss due to the disease or weight gain due to possible hormone therapy. The ROI is composed of many points in the highest resolution forming a surface mesh. Therefore, the 3D surface alignment is more reliable than 3 skin markers, tattoo points, or cast lines on the mask. In addition, the integrity of the 3D surface is more reliable, unlike skin markers/tattoos that can be moved around by stretching or squeezing the local skin at the setup. The cast line on the mask represents the mask position and may only represent the patient head position if the customized thermoplastic mask and headrest fit the patient exactly the same way as in the simulation scan. These conventional methods have been used in the development of frameless SRS procedure^5^ and current HN treatments.^18^

Skin tattoo has been applied for patient setup for radiotherapy treatment as the clinical standard because the tattoos are effective to mark, position, and align the patient using room lasers at both treatment simulation and delivery, so the patient can be set up reproducibly.^18^, ^27^, ^28^ However, it is not desirable from patient’s point of view because of personal preference, comfortability, and religious concern. With the recent advents in SGRT techniques, a tattoo‐free patient setup has been proposed and practiced in some clinics.^29^ Although it is plausible to perform SGRT only, systematic studies on the patient setup accuracy are needed to set the solid foundation for such a paradigm change in the radiotherapy clinic. The SGRT‐only setup is likely to be site specific, depending on the deformability and mobility of the anatomic tissue, and this study provides an initial but direct assessment of the SGRT‐only accuracy in the head region.

Additionally, after the initial patient setup, intrafractional motion monitoring can be readily implemented after capturing an on‐site new reference image, which will reset the residual setup error and only provide motion data during treatment. For the ROI within the open‐face mask at high resolution, the frame rate of the SGRT system is high enough (3–4 Hz in version 5.1 and 8–12 Hz in version 6.2). When the motion management interface (MMI) communication with a Linac machine is enabled, such as the TureBeam system, the radiation beam can be gated with a set motion threshold(s). By doing so, the uncertainty for intrafractional motion is restricted within the clinical threshold. However, IMRT or VMAT treatment with standard fractionation may not need SGRT motion monitoring if the SRS patient immobilization device is applied as the patient motion incident rate is low (<4%) and the magnitude of the motion is relatively small (<3 mm).^30^

### 4.3 Other concerns, limitations, and future directions on SGRT

When using SGRT‐only patient setup and motion monitoring, the patient immobilization device is important, and here, the SGRT‐only setup accuracy is derived from the CDR system with customized head mold and open‐face mask. It has been reported that patient setup accuracy is higher using individual customized head support compared with standard headrest.^31^ In addition, the customized head immobilization system also provides much higher patient motion restriction, reducing possible intrafractional patient motion.

In this study, we also see <4% outliers that have >5 mm SGRT setup differences due to the large vertical skin‐to‐isocenter distance of centralized isocenter. The outliers often have a large SI translational difference and large pitch rotational difference, as shown in Fig. 3. Interestingly, we also observed a mild correlation between the SI translation difference and pitch rotation difference (r = −0.29), suggesting that the 2 differences are related, namely raising the uncertainty in one would result in enlarged uncertainty in the other. Similarly, we observed cases in which the isocenter is near the lateral brain edge and SGRT setups have large LR and Roll differences, which are also mildly correlated (r =−0.44). These cases attribute to the largest differences (outliers) as shown in Table 1. Due to the observed mild correlation between translations and rotations, such as SI‐Pitch (r = −0.29) and LR‐Roll (r = −0.44), the large translational differences may result from the corresponding rotational differences. This suggests that the isocenter (also the rotational center) may have much less difference than appearing at the anterior ROI, as depicted in Figs. 3(a) and 3(c). Nevertheless, this limitation of the SGRT‐only setup can be avoided by screening patient candidates from their plan isocenter location.

Patient weight changes are another important factor that can affect the SGRT‐only setup. Patients under hormone therapy, such as GBM patients, in conjunction with radiotherapy, may not be suitable for SGRT‐only setup, owing to substantial facial swelling, differing from the simulation CT surface. For this group of patients, they should be excluded from the SGRT setups. On the other hand, if a patient experiences substantial weight loss within a week from the simulation, the facial ROI may also be affected. A clinical assessment procedure should be established to identify these patients for exclusion from SGRT setup, including a check on the patient’s weight and concurrent hormonal therapy.

In our clinic, the initial efforts in patient data preparation are made by the dosimetrists: from a planning system to SGRT system and from isocenter check to ROI creation. However, to make the ROI truly patient specific and optimal for SGRT, therapists at the treatment console should be able to exclude deformable skin from the ROI with the guidance of a color‐coded deformation tool. We have trained the therapists to modify the patient‐specific ROI at the treatment for both SGRT patient setup and motion monitoring.

As a continuation to our efforts in patient setup and motion monitoring using SGRT only for radiotherapy of brain and nasopharynx cancer patients with conventional fractionations, we will put efforts to study other anatomical sites to go tattoo free, such as head and neck and breast treatments. For each anatomical site, there are site‐specific concerns and the conclusion from a study on one site may not be directly applicable to another site without additional site‐specific investigation.

## 5 CONCLUSION

In this study, we investigated the SGRT‐only setup accuracy using CBCT as reference. It demonstrated that the SGRT‐only patient setup can be performed not only accurately but also fast inside the treatment room. The SGRT setup differences from CBCT are 1.0 ± 2.5 mm and 0.1˚±1.4˚ on average among 415 treatments of 269 patients and within 5mm/3˚ with 95% confidence level (*P* < 0.001), while the in‐room setup time is usually less than 1 min as capable of real‐time motion monitoring. Therefore, the SGRT‐only patient setup has the potential to be implemented with improved performance in the radiotherapy of brain and nasopharynx cancer patients with standard fractionation.

## Conflict of Interest

The authors have no relevant conflict of interest to disclose.

## Data sharing

For patient setup data used in this study, we will make efforts for anonymization and make them available upon request from readers.

## Author contribution

All authors have made significant contributions to this clinical study to be qualified as coauthors.
